# Plastic Surgeons Defend Textured Breast Implants at 2019 U.S. Food and Drug Administration Hearing: Why It Is Time to Reconsider

**DOI:** 10.1097/GOX.0000000000002410

**Published:** 2019-08-30

**Authors:** Eric Swanson

**Affiliations:** From the Swanson Center, Leawood, Kans.

## Abstract

Textured breast implants were the subject of a U.S. Food and Drug Administration (FDA) hearing on March 25 and 26, 2019. Regulating agencies in other countries, including all of Europe and Canada, have already banned macrotextured implants. Patients affected by Breast Implant-Associated Anaplastic Large-Cell Lymphoma (BIA-ALCL) recounted their life-changing experiences, and requested a ban on textured devices. Plastic surgeons, many with industry ties, spoke in favor of keeping the devices available. The historical advantages of textured implants were presented, including a reduced capsular contracture rate. A 14-point plan to improve sterility at the time of implantation was promoted as an effective alternative to reduce both capsular contractures and BIA-ALCL risk. However, recent studies show that textured implants have not delivered on their early promise. Biocell implants perform worse, not better, than other implant types, and capsular contracture rates are not significantly reduced according to recent core studies. The only known risk factor for BIA-ALCL is textured implants. The lifetime risk for Biocell implants is at least 1:2, 200. There is no reliable evidence that surgical technique makes a difference in risk. This serious issue represents a case study of conflict of interest. In light of recent information, a re-analysis of the true risks and benefits of textured implants is justified. It is time for our professional societies to recognize that the device is the problem rather than surgical technique. On May 2, 2019, the FDA decided against a ban on textured breast implants.

The harrowing testimony of women affected by Breast Implant-Associated Anaplastic Large-Cell Lymphoma (BIA-ALCL) left an impression on those attending the recent U.S. Food and Drug Administration (FDA) hearing.^1^ One courageous young woman showed a photograph of herself with no breasts and a badly scarred chest. Another woman, whose life was saved by brentuximab, told her story of failing six rounds of CHOP (cyclophosphamide, hydroxydaunorubicin, oncovin, prednisone) chemotherapy, a terminal prognosis with 4 to 6 months to live, a stem cell transplant, and a second malignancy related to the stem cell transplant. She said her life as she knew it ceased to exist. She characterized her cancer as “man-made” and “profit-driven.” She said her suffering was compounded by a dismissive industrial public relations campaign, which views her as an anomaly. Another woman, her voice also breaking, told the panel how her treatment has been unsuccessful to date in eradicating metastases and she is fighting for her life, having recently developed a new chest wall mass and a positive positron emission tomography scan despite implant removal, capsulectomies, lymph node and partial rib resection, chemotherapy, and cryoablation. She said that Allergan (Allergan plc, Dublin, Ireland) offered her $7500 if she would never speak about her experience (Allergan requires a general release of liability to issue this payment).^2^ Another woman was informed that her newly diagnosed heart failure was caused by 6 cycles of CHOP chemotherapy. The affected women requested a ban on textured breast implants in the United States. Several women referenced the recent recall of Allergan Biocell textured breast implants in >30 other countries, including all of Europe and Brazil. A webcast of the full hearing is publicly accessible and is recommended viewing for any plastic surgeon who inserts breast implants.^3,4^

Representatives for all 3 of the manufacturers that sell textured implants in the U.S. (Allergan, Mentor, and Sientra) recommended that textured implants remain available.^3^ Plastic surgeons also spoke to the panel.^3,4^ Many were female plastic surgeons, and some had undergone breast augmentation themselves. Remarkably, none called for abandoning textured devices. Several plastic surgeons repeated Adams’ unsupported statement that “technique is critically important,” and requested that the FDA limit breast augmentations to plastic surgeons certified by the American Board of Plastic Surgery.

In his presentation to the FDA, Dr. Adams, lead author of a 2017 study of 42,000 Allergan Biocell implants,^5^ claimed that following the 14-point plan to improve sterility not only reduces the risk of capsular contracture from 50% 3 decades ago to <1% in the past 5 years, but also eliminates the risk of BIA-ALCL.^3^ Adams cited a 9 year mean follow-up, which is shorter than the 11.7 year follow-up reported in the study,^5^ but still quite extraordinary for cosmetic breast surgery patients, who are often lost to long-term follow-up.^3^ In fact, the referenced study reported a 2.2% capsular contracture rate, not <1%.^5^ It is unclear how these spectacularly low contracture rates were determined. Adams stated that all 8 surgeons in the study, none of whom had encountered a case of BIA-ALCL, used the 14-point plan, although whether the 14 points were actually followed for the duration of the study is questionable, along with the retrospective study design, which invites selection bias.^6^ Six authors were Allergan consultants, including Adams, who stated at the hearing that he had no financial disclosures for his presentation, beyond his educational activities.^3^ A recent journal disclosure indicated that he is a consultant, advisor, and research coordinator for Allergan and Sientra.^7^ According to the propublica.org website, Adams received >$75,000 from Allergan in 2016, the most recent year for which payment data are available.^8^ A representative for Allergan also referenced the study by Adams et al,^5^ reiterating such points as changing gloves, antiseptic solutions, minimal touch, and quoting a zero rate of BIA-ALCL when these steps are taken.^3^

This stunning drop in capsular contracture rate, from half of all breast augmentations to almost none, contrasts sharply with data from manufacturer core studies. A 2014 study of Allergan Natrelle silicone gel breast implants with 10-year follow-up indicated a capsular contracture rate of 18.9% for augmentation with no significant difference between textured and smooth devices.^9^ A 2015 core study reported a capsular contracture rate of 9.2% for the Biocell 410 textured implant.^10^ Capsular contracture rates demonstrate no recent downward trend (Fig. 1).^9–23^

**Figure 1.**
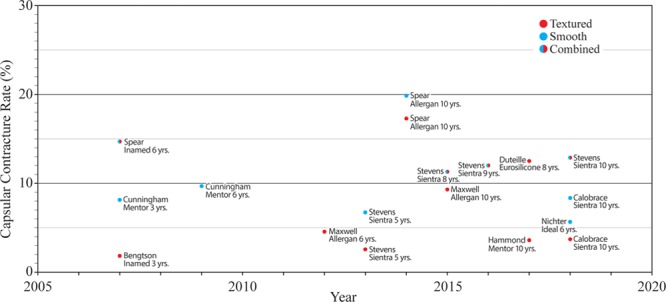
Grade III/IV capsular contracture rates for primary breast augmentation as reported in manufacturer core studies. There is no significant trend.

Several studies published about 4 decades ago reported capsular contracture rates in the range of 27–40% after breast augmentation.^24–29^ This spike in capsular contracture rates was caused by leak-prone thin-shelled second generation implants inserted in the 1970s, which almost always ruptured within a decade of insertion.^30^ Silicone leakage is known to increase the risk of capsular contracture.^31,32^ This unfortunate experience was improved by the introduction of third generation devices in the 1980s, with an additional barrier layer in the shell.^30^ Two meta-analyses published in 2006 found no evidence of a reduction in capsular contracture rates using textured implants when implants are placed subpectorally,^33,34^ the plane preferred by most surgeons today for breast augmentation.

In his FDA presentation, Clemens reaffirmed the remarkable fact that there has been no case published of BIA-ALCL occurring in a woman implanted only with smooth implants, whose implant history is fully documented.^3^ Clemens challenged the scientific foundation for the 14 points, noting that none of these points have been linked scientifically to BIA-ALCL risk.^3^ The problem was unknown before textured implants were introduced. Indeed, a simple 1-point plan to abandon textured devices can be expected to eliminate this serious health risk.^6^

Textured implants were initially designed to reduce the risk of capsular contracture.^33,34^ Shaped, textured implants have been promoted to produce a more natural “teardrop” breast shape (Fig. 2).^15,35^ In women undergoing breast reconstruction, shaped implants may be used to preferentially fill out the lower poles.^36^ With the advent of shaped form-stable “gummy bear” implants, surface texturing was used in an effort to fix the implant to adjacent breast tissue and prevent movement.^12,35^ Many plastic surgeons believe that shaped implants produce a superior aesthetic result,^15^ especially in women with constricted or tuberous breasts.^36^

**Figure 2.**
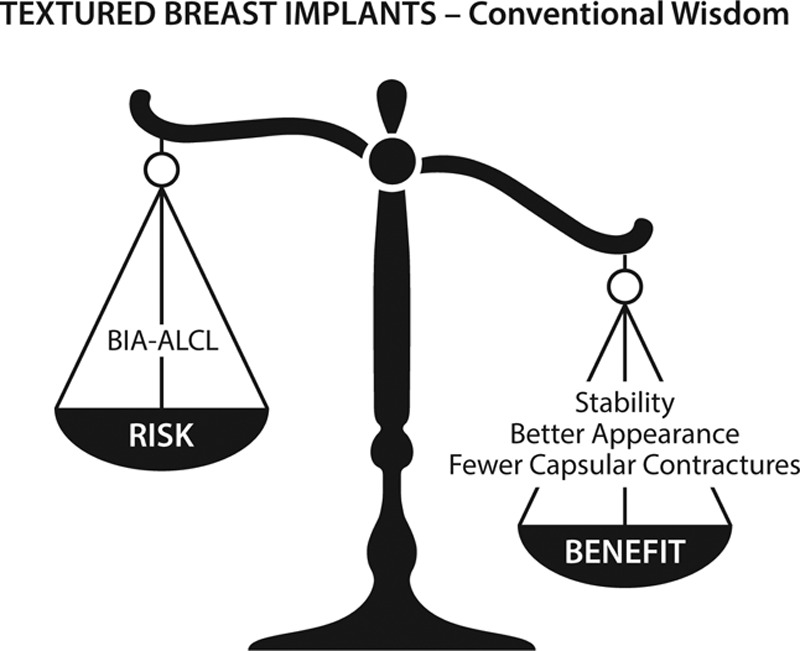
Risks versus benefits for textured breast implants, as originally conceived.

Recent studies, however, reveal that texturing has not delivered on its promises.^37–39^ Hall-Findlay was the first to raise concerns regarding late seromas and double capsules.^37^ In 2018, van Slyke et al reported that Biocell implants are not superior in their performance, but rather alarmingly inferior compared with other implant types. Deficiencies in performance included the shortest time to explantation, implant malposition, malrotation, seroma, rippling, capsular contracture, rupture, pain, and double capsules.^38^ A 2017 ultrasound study by Sieber et al revealed that textured, shaped implants rotate in their pockets in 42% of cases.^39^ Indeed, there is no evidence that texturing prevents implant movement as is often claimed, bringing into question the value of texturing in the first place. Figures 2 and 3 compare the risks and benefits of textured versus smooth implants.

Unfortunately, after listening to Dr. Adams’ presentation, and others, the FDA panelists might reasonably conclude that textured implant surfaces almost eliminate capsular contracture, and that BIA-ALCL does not occur when the 14 points are followed. In their open deliberations, the FDA panelists quite reasonably sought to balance the benefit versus risk. Dr. Lewis, the panel chairperson, noted the difference in risk among types of texture and that the available data may be sufficient to conclude that a heightened risk is associated with highly textured implants.^3^ Dr. McGrath, a panelist, disagreed, warning that abandoning textured implants is likely to cause a “tsunami” of reoperations and force plastic surgeons to wrap implants in mesh, such as the costly acellular dermal matrix, which poses additional risks.^3^ There was no recognition that many plastic surgeons have already made the transition to using smooth implants exclusively,^40^ both for augmentation and reconstruction, with no reported increase in complications and without wrapping all implants in mesh.

One FDA panelist asked, what is the denominator? Clemens reported the most reliable risk estimate in patients implanted with Biocell 410 devices, which stands at a 1:2200 lifetime risk according to a prospective study by McGuire et al,^35^ supplemented by 4 additional cases of BIA-ALCL diagnosed after publication.^3^ Importantly, McGuire has abandoned Biocell 410 implants.^41^ The denominator and numerator are clear – 17,656 women, 8 cases of BIA-ALCL (and likely to increase over time).^3^ According to information presented by manufacturers to the panel, smooth implants now represent about 90% of the 300,000 breast augmentations performed each year in the United States.^3^ The denominator of women with smooth implants is therefore enormous, millions of women worldwide, with no cases of BIA-ALCL yet documented in a woman known to have received only smooth implants, making the numerator zero.^3^ (The significance of this remarkable fact does not change if BIA-ALCL is eventually diagnosed in a woman treated only with smooth implants, as long as the risk remains minuscule compared with textured devices.)

The appropriate course of action is clear once the facts are known:

1. Textured, and especially Biocell, implants are linked to BIA-ALCL.
2. Smooth implants are not linked to BIA-ALCL.
3. Textured implants are not superior or equivalent to smooth devices, but rather inferior.
4. There is no reliable evidence that the 14 points eliminate BIA-ALCL risk.
5. A diagnosis of BIA-ALCL causes unnecessary morbidity and expense to women, even if it is seldom fatal.

As an ethical matter, and one consistent with the basic principle of “primum non nocere,” Biocell implants should be removed from the market to eliminate this serious health risk.^42^ The French regulatory authority came to this conclusion despite the popularity of textured implants in Europe.^43^ Shortly after the FDA hearing, Canada banned macrotextured breast implants.^44^

It is often stated that, if diagnosed early, the prognosis for BIA-ALCL is excellent.^3,4^ However, in many cases (15%), the diagnosis is delayed, and the cancer has spread beyond the capsule, requiring adjuvant chemotherapy, immunotherapy, or radiation.^3^ The diagnosis and treatment may be delayed for a variety of reasons, including accessibility of quality healthcare and patient concern regarding the cost of a diagnostic workup. One patient told the panel that treatment of BIA-ALCL cost her $288,000.^3^ Many women have suffered severe financial hardship because of this diagnosis.^3,4^

In post-hearing emails to members of our societies, all 3 manufacturers of textured implants defended their products. Allergan emphasized that the benefits of breast implants outweigh the risks. Of course, the word “textured” is conspicuously absent in the communication. Few deny that the overall benefits of *smooth* implants outweigh the risks (notwithstanding the issue of Breast Implant Illness). Allergan disagrees with the French regulatory action and reassures the public that there is no “immediate” risk.^43^ Of course, the concern is the long-term risk.

An unstated reason for keeping textured devices available is that plastic surgeons and manufacturers do not wish to see a ban on textured implants because such an action may cause a groundswell of women who want their implants removed or replaced.^45^ This reaction is no different from an automobile manufacturer resisting acknowledging a problem because of the expense of recalling millions of vehicles. Unfortunately, regulators are often needed to force companies to take the appropriate action. Potochny et al^45^ found that, once informed, a minority of women (3.4%) return to have their implants removed or replaced. Women should not be reassured that there is no reason to replace their implants. That decision is for them to make.

An analogy is to be found in the recent reaction to the tragic Boeing 737 MAX crash in Ethiopia. The manufacturer initially stood by the safety of the aircraft despite 2 eerily similar crashes. Boeing insisted the aircraft was safe to fly even with the software problem uncorrected. On the day after the Ethiopian crash, the Federal Aviation Administration announced that their review showed “no systemic performance issues and provides no basis to order the grounding of the aircraft.”^46^ However, other countries quickly grounded the airplanes. The Federal Aviation Administration followed suit after international pressure. In hindsight, the decision to ground the airplanes until the software problem is corrected appears obvious. Notably, Boeing initially blamed the pilots for the crashes, not the airplanes.^46^ In the case of breast implants, the manufacturers and industry-funded investigators blame plastic surgeons for inadequate sterility rather than a faulty device.^42^

The FDA makes a point to hear from all stakeholders.^3^ Many plastic surgeons who attended the hearing have financial ties with the manufacturers. Some surgeons have staked their reputation on them. Plastic surgeons who have abandoned textured implants may see no reason to attend and voice their opinion; they do not plan to use textured implants regardless of the FDA decision. The same day of the hearing, NBC News aired an interview with 8 BIA-ALCL patients, all of whom recommended a ban, and one patient’s plastic surgeon, who said that he would never again implant a textured device.^47^ The implanting surgeons were certified by the American Board of Plastic Surgery or the Royal College of Surgeons of Canada.

A common theme is patient choice.^3,4^ Some surgeons recommended that textured implants remain available so as not to deny women their right to choose. Implant choice is highly influenced by the advice of plastic surgeons. Few, if any, women would choose textured devices if they are properly informed of the true risks and benefits (Figs. 2 and 3). Patients should not be offered an unsafe treatment option.

One question underscores the contradiction in recommending textured breast implants. If texturing is not really the problem, why is it universally recommended^48^ that women who develop BIA-ALCL and desire implant replacement be given smooth implants? It is reasonable for patients to ask, if smooth implants are acceptable for repeated breast augmentation, why were they not used initially? Remarkably, Lamaris et al^48^ recommend that surgeons only implant smooth implants for breast reconstruction in women affected by BIA-ALCL, not because textured implants are the problem, but rather because of a genetic predisposition. Denial is in full force.

We cannot rely on the legal system for a remedy because of preemption.^49^ Class 3 devices are protected from patient lawsuits. If this were not the case, textured implants would have been removed from the marketplace years ago to avoid product liability claims.

At the FDA hearing, and in the NBC news report,^47^ the implant manufacturers assured the public that patient safety comes first.^3^ However, this affirmation rang hollow to many women in the audience.^3^ Talking points circulated to plastic surgeons includes the sentence, “available data does not support discontinuance of textured implants.”^50^ The International Society of Aesthetic Plastic Surgery released an announcement condemning the French ban. A “best practices” statement from our societies calls for improved sterility instead.^51^ Regrettably, our plastic surgery societies and journals have been misled by some conflicted “thought leaders.”^42^ How did plastic surgeons get to this point, defending a false narrative (a faulty technique, not device), and speaking with one voice on behalf of the manufacturers? This subject is a strong testament to the power of financial conflict of interest in our specialty.^42,52^ It is time for our societies to “unendorse” the 14-point plan and recommend against the continued use of this harmful product.^42^

On May 2, 2019, the FDA decided against banning textured implants.^53^ In support of this decision, the FDA noted that although a “majority” of women with BIA-ALCL had textured implants, there are known cases in women with smooth implants. This surprising conclusion is at odds with Dr. Clemens’ testimony to the panel that there are no published cases occurring in women treated with smooth implants whose implant history is fully known.^3,54^ The FDA also noted that <10% of breast implants sold in the U.S. are textured.^53^ It is unclear, from an ethical standpoint, how the percentage of women implanted with this device is relevant to the decision. The number of deaths from BIA-ALCL jumped from 19 reported mortalities before the FDA hearing to 24 fatalities 1 month after the hearing.^54^ Whatever one’s opinion of textured breast implants, there can be little doubt that without them, most of these women would be alive today.

**Figure 3.**
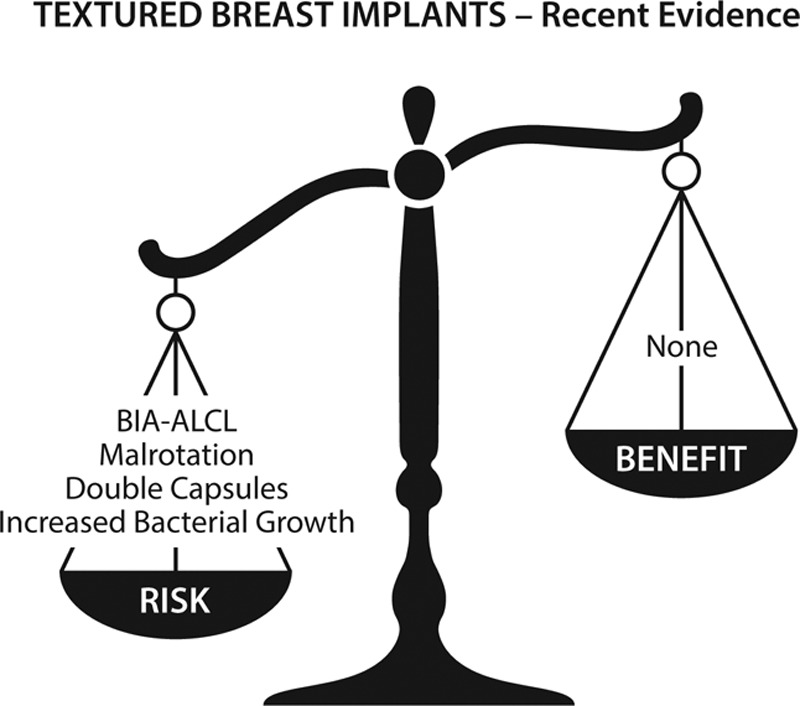
Risks versus benefits for textured breast implants, according to recent studies.
